# Antimicrobial Potential of the Genera *Geobacillus* and *Parageobacillus*, as Well as Endolysins Biosynthesized by Their Bacteriophages

**DOI:** 10.3390/antibiotics11020242

**Published:** 2022-02-12

**Authors:** Joanna Zebrowska, Małgorzata Witkowska, Anna Struck, Patrycja E. Laszuk, Edyta Raczuk, Małgorzata Ponikowska, Piotr M. Skowron, Agnieszka Zylicz-Stachula

**Affiliations:** Department of Molecular Biotechnology, Faculty of Chemistry, University of Gdansk, 80-308 Gdansk, Poland; joanna.zebrowska@ug.edu.pl (J.Z.); mwkbm2017@gmail.com (M.W.); anna.struck@phdstud.ug.edu.pl (A.S.); patrycja.laszuk@phdstud.ug.edu.pl (P.E.L.); edyta.raczuk@phdstud.ug.edu.pl (E.R.); malgorzata.ponikowska@phdstud.ug.edu.pl (M.P.); piotr.skowron@ug.edu.pl (P.M.S.)

**Keywords:** *Geobacillus*, *Parageobacillus*, antimicrobial compounds, antimicrobial potential, candidate probiotics, bacteriocins, endolysins

## Abstract

In the recent decades, antibiotic resistance has emerged and spread rapidly among clinically relevant pathogens. The natural ability of bacteria to transmit resistance determinants through horizontal gene transfer poses constant challenges to drug development. Natural molecules produced by soil microorganisms continue to be a key source of new antimicrobial agents. In this context, bacteria from the *Geobacillus* and *Parageobacillus* genera deserve special attention. Although there is commercial and industrial interest in these microorganisms, the full range of antibacterial compounds biosynthesized by the *Geobacillus* and *Parageobacillus* species remains largely unexplored. The aim of this review is to present the strong antimicrobial potential of these bacteria and endolysins produced by their bacteriophages.

## 1. Introduction

**Figure 1:**
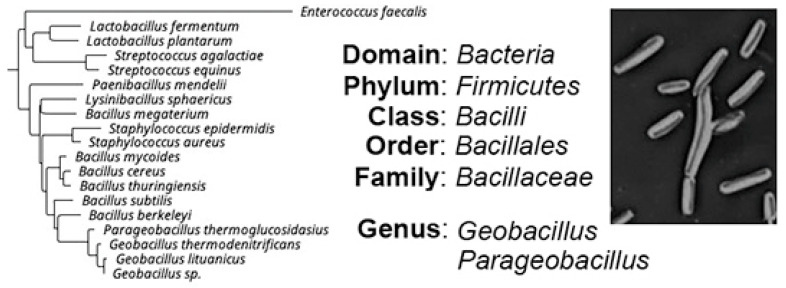
Current taxonomic classification of the genera *Geobacillus* and *Parageobacillus*.

*Geobacillus* and *Parageobacillus* genera belong to the *Bacillaceae* family, which is a large and heterogeneous group that includes many mesophilic, facultative thermophilic and thermophilic species, widely distributed in different habitats [1]. The current taxonomic classification of *Geobacillus* and *Parageobacillus* is presented in Figure 1. Their closest evolutionary relatives are bacteria of the genus *Bacillus*, exemplified by *Bacillus subtilis* (*B. subtilis*) [1]. *B. subtilis* strains and other species from the genus *Bacillus* are well known for their probiotic properties and production of various antimicrobial compounds [2,3,4].

*Geobacillus* and *Parageobacillus* have been found in various locations, differing geographically and environmentally. They have been detected on all continents and also in the seas, oceans and even in the atmosphere [5,6]. Representatives of these genera can be isolated from extreme places such as hot springs, hydrothermal vents, high-temperature oil fields, composts, and greenhouse soil [1,6]. Interestingly, they have also been found (in large numbers) in cold places, much below their minimal growth temperatures, such as ocean sediments or soil samples [6].

The genera *Geobacillus* and *Parageobacillus* comprise thermophile, endospore-forming, chemo-organotrophic rods. The structure of their cell walls is Gram-positive, but the result of the gram-stain reaction may be varied. Cells are motile or non-motile and can occur individually or in chains [1,5,7]. Cellular fatty acids are characterized by iso-15:0, iso-16:0 and iso-17:0. The G + C content of DNA ranges between 48.2 and 58 mol% [7]. Depending on the strain, these bacteria are aerobic or facultatively anaerobic. Their growth is observed in a pH range between 6.0 and 8.5 and temperature range of 35–80 °C (optimum 55–65 °C) [1,6,7].

Until 2001, *Geobacillus* and *Parageobacillus* were classified on the basis of 16s rRNA gene sequence analysis as thermophilic variants of *Bacillus* spp. [8]. The genus *Bacillus* initially comprised five phylogenetically distinct groups, with the future *Geo*- and *Parageobacillus* included in group 5 [8]. Subsequently, according to physiological characteristics, 16S rRNA gene sequences analyses, fatty acid composition analyses, G-C contents and DNA–DNA homology studies, *Geobacillus* and *Parageobacillus* were reclassified together as *Geobacillus* gen. nov. [7]. In 2016, Aliyu and colleagues separated *Parageobacillus* from *Geobacillus* [9]. In their research, they used multiple phylogenomic strategies to estimate relatedness between sixty-three *Geobacillus* strains, whose genome sequences were available at the time. Their analysis allowed them to distinguish two clades on the basis of differences in nucleotide base composition. Clade I with G + C content 48.8–53.1% (*Geobacillus* species) and clade II with 42.1–44.4% (new, *Parageobacillus* species). Species belonging at the baseline of *Geobacillus*, which have been moved to *Parageobacillus*, include: *P. caldoxylosilyticus*, *P. thermoglucosidasius*, *P. thermantarcticus*, *P. toebii* and *P. genomospecies* 1 (NUB3621) [9]. Subsequently, in 2020, Najar and colleagues presented the analysis that supports their proposal of including two other species: *G. galactosidasius* and *G. yumthangensis* in the genus *Parageobacillus* [10].

As genera derived from extreme environments, *Geobacillus* and *Parageobacillus* are sources of proteins that are stable at high temperatures and are functional under other extreme conditions, especially compared to mesophilic homologues. Over the past few years, an extensive exploration of the *Geobacillus* and *Parageobacillus* transcriptomes and secretomes has revealed many proteins with either proven or potential industrial and medicinal applications [1,11,12]. The strong metabolism and cellular propagation of these organisms make them appropriate hosts for different bioprocesses (whole-cell applications) [5]. Thus, these bacteria are exploited in various biotechnological and industrial applications, such as food production, the textile industry, the paper industry, bio-detergent technology, cosmetics, drugs and the pharmaceutical industry, biofuel or chemical production, bioremediation and many others [6,13,14]. Recently, *Geobacillus* has been investigated as a source of thermostable L-asparaginase with potential therapeutic properties [15]. There are also promising reports about the antimicrobial applications of *Geobacillus*. Alkhalili and colleagues describe *Geobacillus* sp. strain ZGt-1, isolated in Jordan, which demonstrated an antimicrobial activity against *G. stearothermophilus*, *B. subtilis* and *Salmonella typhimurium* (*S. typhimurium*) [16]. Additionally, Pokusaeva and colleagues reported that they had identified and partially purified bacteriocins (ribosomally produced proteins that inhibit other strains or species) synthesized by *G. stearothermophilus* strains isolated from oil-wells in Lithuania [17]. Such research may indicate many new valuable applications for the genera *Geobacillus* and *Parageobacillus*.

## 2. Antimicrobial Potential of *Geobacillus* and *Parageobacillus*

### 2.1. Geobacillus and Parageobacillus as a Source of Novel Antimicrobial Compounds

**Figure 2:**
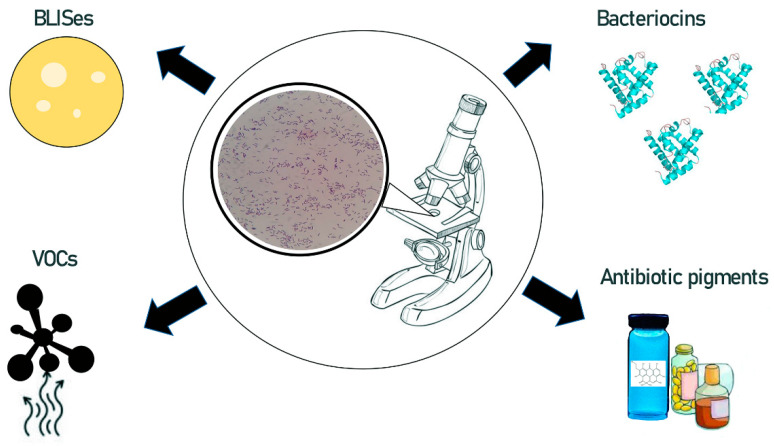
Types of *Geobacillus* and *Parageobacillus* derived antimicrobial compounds.

The potential of *Geobacillus* and *Parageobacillus* to produce a wide range of bioactive metabolites (that mediate antibiosis) is not fully explored. In this section we list, and briefly characterize, the antimicrobial molecules produced by *Geobacillus/Parageobacillus* that have been described in the literature so far. Types of the *Geobacillus* and *Parageobacillus* antimicrobial compounds are exemplified by several bacteriocins, bacteriocin-like inhibitory substances (BLISes), volatile organic substances (VOCs), and antibiotic pigments (Figure 2).

#### 2.1.1. *Geobacillus*-Derived Volatile Organic Substances and Antibiotic Pigments

*Geobacillus* sp. (M-7) appears to have a unique approach to combatting competitive bacteria by producing a range of volatile organic substances (benzaldehyde, acetic acid, amongst others). The *Geobacillus* strain, as well as a mixture of the detected chemicals, was able to inhibit the growth after 48 h and cause death after 72 h of *Aspergillus fumigatus*, *Botrytis cinerea*, *Verticillium dahliae* and *Geotrichum candidum* [18].

*Geobacillus* sp. Iso5 produces a unique cyanoxanthomycin-type antibiotic pigment. This fluorescent pigment shows potent antimicrobial activity against selected Gram-positive and Gram-negative bacterial species: *B. subtilis* (MTCC 3053), *E. coli* (MTCC 1698), *Pseudomonas aeruginosa* (*P. aeruginoasa*) (MTCC 6458), *Staphylococus aureus* (*S. aureus*) (MTCC 6908) and *Streptococcus* sp. (MTCC 9724) [19].

*Geobacillus* sp. LEMMJ02 is a thermophilic bacterial species isolated from the sediments of an Antarctic volcano on Deception Island. Annotation of the *Geobacillus* sp. LEMMJ02 genome revealed the presence of genes associated with the production of secondary metabolites with antimicrobial properties. The strain is likely to produce phengycin (antifungal lipopeptide), bacteriocin, and terpene [20].

#### 2.1.2. *Geobacillus* and *Parageobacillus* Derived Bacteriocins

Bacteriocins are peptides with antimicrobial activity. They are synthesized by many bacteria and archaea strains. Various types of these molecules have been identified and purified since their discovery in 1925 by André Gratia [21]. 

The synthesis of bacteriocins is carefully regulated. Bacteriocins are synthesized by the ribosome in an inactive form. Posttranslational modifications and cleavage of the leader sequence help the cell to avoid self-inflicted damage. Specific defense proteins and efflux pumps are also employed. Bacteriocins are secreted during the logarithmic growth phase of bacteria. Their secretion is stimulated by various environmental factors, including bacterial cell density, nutrient availability, the presence of acetic acid, and signal peptides such as competence-enhancing peptides [22,23]. For example, *Firmicutes* commonly regulate the expression of the bacteriocin encoded genes through quorum sensing [24].

Bacteriocins are a very broad group and are classified into several different families, hence their diverse mode of action and a wide variety of affected strains [23,25]. The growing interest in exploiting the potential of these molecules led to the development of databases and search engines capable of identifying plausible bacteriocin genes and providing information about already characterized species [26,27,28,29,30].

Currently, bacteriocins are used mainly in the food processing industry as food preservatives. Nisin is one of many bacteriocins derived from lactic acid bacteria (LAB)-*Lactococcus lacti.* It is a lantibiotic—a polycyclic peptide with incorporated lanthionine and methyllanthionine and antimicrobial activity [31]. It is legally approved as a food preservative and was also investigated for its biomedical applications. Some bacteriocins are capable of killing bacterial spores [32]. More recently, the effects of bacteriocins on fungi and pathogenic bacteria, including antibiotic-resistant strains, were studied [33,34,35,36,37,38]. Some bacteriocins were found to affect specific bacterial strains; therefore, it is possible to use them for treatments on human microbiomes including the gut microbiome [39,40].

Thermophilic bacteria from the genus *Geobacillus* and *Parageobacillus* are a source of vital enzymes, and widely employed in biotechnology and industry. As most bacteriocins are derived from Gram-positive bacteria, *Geobacillus* and *Parageobacillus* can be also considered as a rich source of such antimicrobial peptides. 

There are already several characterized and semi-characterized bacteriocins derived from *Geobacillus* and *Parageobacillus* strains (Table 1). However, their modes of action and mechanisms of secretion have not been thoroughly investigated. The two most investigated—geobacillin I and geobacillin II—are nisin analogs. Their N-terminal structure is very similar to that of nisin rings but the C-terminal sites show no homology. The mode of action of geobacillin I, similarly to nisin, includes the formation of the pores in the cell membrane [41]. Geobacillin I and geobacillin II were identified in the genome of an oil-well-derived strain *G. thermodenitrificans* NG-80-2 [42]. Several other strains were confirmed to produce geobacillin I. Geobacillin I showed a comparable bioactivity range to nisin but higher stability in the pH 7 and 8 range at 37 °C and 60 °C. Recombinant geobacillin II showed antimicrobial activity only towards *Bacillus* species [42,43].

Several *Geobacillus* spp. were tested for bacteriocin activity only against closely related strains, for example: *G. stearothermophilus* 15 [51], *G. stearothermophilus* 31 [17], *G. stearothermophilus* NU-1, NU-2, NU-4, NU-7, NU-23W, NU-34, NU-37, NU-41, NU-44, NCA-1373, NCA-1492 [52], *G. stearothermophilus* DSM 458 [53]. Further testing of these compounds might reveal their utility in the fight against pathogenic and antibiotic-resistant strains. Such an investigation of thermocin from strain *G. stearothermophilus* NU-10, previously reported to be active against other *Geobacillus* strains [52], led to the identification of thermocin 10 activity against *B. circulans* 4516 [48]. Strong proof of this concept was observed in the study of Pranckutė et al. Screening of 101 strains of *Geobacillus* revealed that each of them produces bacteriocins active against other *Geobacillus* spp. bacteria. Moreover, all the investigated strains were able to inhibit the growth of at least one pathogenic strain (out of the 19 tested) [50].

An in silico search for novel bacteriocins, employing genome mining tools, resulted in the identification of several putative *Geobacillus* bacteriocins and their genetic structures [53,54,55,56,57]. These findings highlight the potential, which is yet to be revealed for *Geobacillus* and *Parageobacillus*, as a source of antimicrobials.

#### 2.1.3. *Geobacillus* and *Parageobacillus* Derived Bacteriocin-like Inhibitory Substances

BLISes are bacteriocin-like inhibitory substances, though the line between the bacteriocins and BLISes is blurred. Mostly, BLISes are classified as inhibitory bacterial substances, active against a wider spectrum of microbes than bacteriocins, while also being proteinaceous [58,59]. Though there are precious few articles on the matter, *Geobacillus*- and *Parageobacillus*-derived BLISes are still worth discussing.

Turkish thermal springs/soils delivered the *G. toebii* HBB-247 strain. A 38 kDa BLIS from *P. toebii* was found to be stable up to 60 °C but also susceptible to proteolysis. It has shown bioactivity towards the growth inhibition of several strains including *G. stearothermophilus* (DSMZ 22), *Listeria* sp. (food isolate), *Enterococcus faecalis* (*E. faecalis*) (ATCC 51299), *Enterococcus avium* AS-3, *Anoxybacillus* sp. HBB-134, *Geobacillus* sp. HBB-269, *Geobacillus* sp. HBB-270, *Anoxybacillus* sp. HBB-229, *Clostridium pasteurianum* (DSM 525) and *Cellulomonas fimi* (DSM 20114). Its activity against *C. pasteurianum* (DSM 525) was as high as against *G. stearothermophilus* (DSMZ 22), which adds a point for classifying this antimicrobial agent as a BLIS rather than bacteriocin [60].

*G. stearothermophilus* 32A, 17, 30 and 31 strains, obtained from oil wells in Lithuania, were evaluated for their antibacterial activity and while their prevalent inhibition was centered around various *Geobacillus* strains, their activity was also recorded for three pathogenic microorganisms: *B. cereus* DSM 12001, *Staphylococcus haemolyticus* (*S. haemolyticus*) P903, as well as *P. aeruginosa* ATCC 27853 where there was observed a slight inhibition, by no more than two of the studied strains, in varied combinations. This brings back the question of the already mentioned artificial classification, which distinguishes between bacteriocins and BLISes. The range of organisms affected by the 6–7.5 kDa proteins produced by the considered strains is unquestionably beyond the scope of the bacteriocin definition and, yet the efficacy is in comparison much weaker than against the related *Geobacillus* strains [17].

The *G. pallidus* strain SAT4, found in a Pakistani desert, was reported to produce a polypeptide secondary metabolite of most significant antagonistic activity against *Micrococcus luteus* ATCC 10240, *Staphylococcus aureus* (*S. aureus*) subsp. *aureus* Rosenbach ATCC 6538, *B. subtilis* NCTC 10400 and *P. aeruginosa* ATCC 49189 at 55 °C, with a noted activity at as low as 45 °C and as high as 60 °C, as expected for a thermophilic bacterial strain [61].

A study of the Jordan Zara hot spring *Geobacillus* sp. ZGt-1 revealed a thermostable 15–20 kDa peptide of growth inhibition properties against not only *G. stearothermophilus* strain 10, but also against mesophilic *B. subtilis* and *S. typhimurium* CCUG 31969 grown at 37 °C, while no inhibition effect was found for *E. coli* 1005, *S. aureus* NCTC 83254, *S. epidermidis* or *Proteus vulgaris* [16].

#### 2.1.4. Challenges in the Use of *Geobacillus*/*Parageobacillus* Bacteriocins and BLISes

One of the main challenges in the use of bacteriocin/BLISes seems to be the appearance and transfer of potential bacteriocin resistance. Bacteria can develop antibiotic or bacteriocin resistance through spontaneous mutations in their DNA or horizontal gene transfer. It is believed that bacterial strains can even reach a state of dual resistance for both types of antimicrobial compounds. Additional limitations to the wide use of bacteriocins/BLISes may be their non-specificity or narrow spectrum of activity. However, they can be highly effective when used together with antibiotics in synergistic therapies to improve efficacy and to minimize antibiotic concentrations. A route of bacteriocin administration, especially oral or intravenous, can also pose a problem. Most bacteriocins and BLISes are smaller than 10 kDa and easily degraded by proteases. This leads to their poor bioavailability [23,62]. The short plasma half-life of bacteriocins/BLISes causes the necessity of bioengineering their properties. Moreover, the synthesis of these compounds is inefficient and depends on many conditions. For that reason, research should aim at increasing their synthesis efficiency and improving their stability. Other problematic issues concerning bacteriocins are: (*i*) high production cost, (*ii*) complicated purification process, (*iii*) route of administration, (*iv*) low solubility, (*v*) fast biotransformation, and (*vi*) low half-life. Many aspects of bacteriocin effectiveness are not yet well understood [23,62]. *Geobacillus/Parageobacillus* bacteriocins/BLISes withstand a wide range of pH and temperatures. Thus, they are naturally more stable when compared to bacteriocins derived from mesophilic strains. It is also worth mentioning that their spectrum of activity is quite narrow, which can be either a challenge or an advantage, depending on the potential use. Hopefully, future investigation and usage of the *Geobacillus/Parageobacillus* derived bacteriocins and BLISes will help overcome some of the challenges described above.

### 2.2. The Potential of Geobacillus and Parageobacillus as Probiotics

The probiotic definition has evolved through the years [2,63]. The most commonly used definition is based on the International Life Sciences Institute–Europe and World Health Organization (WHO) work and states that probiotics are “live microorganisms which when administered in adequate amounts confer a health benefit on the host” [2,64,65]. In some countries, including Japan, a different definition is used. The term probiotic includes not only viable microorganisms but also cells of non-viable microorganisms that confer health benefits [63,65]. It is now understood that microbial viability is not always directly related to their culturability.

The probiotics are often antagonistic against other microorganisms. They are believed to exert their effects through the secretion of vitamins, exoenzymes or bacteriocins, among others. Scientific research into probiotics has recently made significant advances and the scale of the probiotic market is constantly expanding. The use of probiotics has significantly increased, especially in food animal production [66,67,68]. Many studies have shown that probiotics improve growth outcomes, survivability, and resilience to diseases [66,68,69]. Many members of the phylum *Firmicutes* have been demonstrated as beneficial microbes used as bimodal probiotic microbiota for humans and animals [2,70].

In a post-antibiotic era, and during a time of growing concerns about antimicrobial resistance, there is a constant need for new candidate probiotics, exhibiting greater inhibitory activity against pathogenic bacteria. Since *Geobacillus* and *Parageobacillus* also belong to the phylum *Firmicutes* (Figure 1), they may provide a rich source of novel candidate probiotic strains with unique properties [71]. These bacteria are known to biosynthesize several antibacterial compounds, such as antibiotics, bacteriocins, and BLISes (see Section 2.1). For example, it was demonstrated that *G. thermoleovorans* could inhibit the growth of some reference pathogenic strains, including *S. typhimurium* (ATCC1408)*, Vibrio parahaemolytiticus* (ATCC 17802), *Vibrio alginolyticus* (ATCC 17749)*, S. aureus* (ATCC 25923) and a β-lactamase-producing *E. coli* strain (ATCC 35218) [45,71]. Additionally, some *Geobacillus*/*Parageobacillus* species are able to interfere with quorum sensing in Gram-negative bacteria [70,72]. Last but not least, their adherence ability to abiotic surfaces may help these bacteria to eliminate potential pathogens and create a bio-secure environment, preventing the introduction and/or spread of harmful microorganisms [71].

Due to the above-mentioned properties, *Geobacillus* and *Parageobacillus* should certainly be considered as beneficial candidate environmental probiotics. Their advantage over other probiotic strains could be their high survival potential. They are capable of surviving in harsh conditions, such as: draughts, UV radiation, extreme pH and temperatures, organic and inorganic chemicals, salts, detergents and the presence of heavy metal ions [73]. In fact, their spores, derived from terrestrial soils, can persist through cold, extreme aridity and UV radiation at high altitudes. Thus, they can be carried in the atmosphere and deposited far from their origins [6,74]. According to Nicholson’s estimation, the longevity of *Geobacillus* spores at 40 °C could be 1.9 billion years [6,75]. Moreover, these non-pathogenic bacteria should pose no risk to humans or animals, as they constitute an essential part of the microbial soil community. For many years, both people and animals have had constant exposure to the soil and the aerosoled dust, and a large variety of microorganisms (or their spores), which originally inhabit soil [74]. They even ate soil. According to archeological evidence, *Homo erectus* and other hominids were already practicing geophagy [74]. It is also worth noting that most primate species are geophageous [74,76]. Nowadays, soil and its microbial components still can enter humans and animals through direct or indirect pathways. Accidental geophagy occurs when improperly washed vegetables are eaten. Contemporary geophagy is defined as willful ingestion of specific types of soil for their nutritive or medicinal value. In several regions of Africa, Asia, South and Latin America, and the pacific Islands, geophagy still remains common practice, among pregnant and nonpregnant women and even men [74,77,78,79]. Interestingly, consumption of soil is seen by geophagic people as a natural stimulant, having a positive and euphoric effect on humans, easing their upsetting thoughts, helping them to relax and struggle with stress [79]. Golokhvast et al. demonstrated that experimental geophagy resulted in significant improvement in the behavior of laboratory rats, subjected to instrumental stress [80]. At this point, it is also worth recalling an intriguing environmental microbiome hypothesis, proposed by Blum et al. [81]. According to this hypothesis, there is a close link between the human intestinal and soil microbiomes. This linkage was established during evolution and is still developing. Both microbiomes can be regarded as “superorganisms” that complement each other with inoculants, genes and growth-sustaining molecules [81]. Extensive research into the geographical and functional links between soils, plant and gut microbiota could benefit human health, sustainable agriculture as well as food industry [81,82].

However, there is another side to the coin: improper selection and inadequate colonization of probiotics may lead to a change in the particular microbiome, not its protection and support [2,83]. Additionally, environmental probiotics with a high survival potential may be difficult to remove if necessary. Thus, the probiotics should be always used with caution. To overcome the technological, economic, and clinical problems, which can be associated with probiotics, scientists adopted postbiotics as a no-viable form. Since postbiotics do not include live microorganisms, the risks associated with their use are minimized [84].

### 2.3. A Fusion and Display System Based on Proteins Derived from Geobacillus

Both *Geobacillus* and *Parageobacillus* genera are known to be a rich source of thermostable, biotechnologically useful proteins. An interesting example of such molecules is a multimeric enzyme—a pyruvate dehydrogenase (PDH)—derived from *G. stearothermophilus*. This multienzyme complex catalyzes the oxidative decarboxylation of pyruvate, which results in acetyl coenzyme A biosynthesis [85,86]. The PDH is composed of three different subunits: E1, E2 and E3. One of the PDH subunits—dihydrolipoamide acetyltransferase (E2 subunit)—has a unique ability to self-assembly into trimers, which integrate into a 60-meric spherical protein cage (molecular mass 150 MDa). Both the E1 (150 kDa) and E3 (90 kDa) subunits are noncovalently bound into the scaffold formed by E2 and are displayed peripherally in the number of 60 copies [87,88,89,90]. The denatured E2 protein can be renatured in vitro, without the participation of chaperonins, to form a catalytically active 60-mer structure with icosahedral symmetry [91,92,93].

Rationally designed, recombinant proteins, peptides or antigens can be attached to the N-terminal end of the E2 polypeptide or can be displayed on its surface in place of the lipoyl domains. The obtained, recombinant E2 fusion protein variants still have the ability to self-assemble into the spherical protein cage structure [87,88,94]. Moreover, the sizes of the recombinant fusion partners are not limited to small proteins or short peptides.

These unique properties of the dihydrolipoamide acetyltransferase were used to obtain a modified E2 variant with an increased number of phenylalanines per subunit. The modified scaffold served as a nanocarrier for Doxorubicin delivery to human breast cancer cells [95].

Additionally, the recombinant Env V3 or Gag(p17) proteins, derived from HIV-1, were fused to the E2 domain and used for vaccine development. The obtained multimeric structures induced T cell response in mice [87,96,97,98]. The modified E2 cage protein was also used for the construction of biosensors, which were able to detect HeLa cells or to measure the concentration of thrombin. The biosensors were obtained by the modular conjugation of the E2 nanoparticles with various functional moieties, enabling antibody, enzyme, DNA aptamer, or fluorescent dye immobilization [99].

The E2 fusion and display system creates opportunities to develop novel applications in biotechnology and medicine, such as new vaccines [100]. The modified E2 scaffolds also have a potential to be used as nanocarriers in antimicrobial therapies, by fusion or encapsulation with recombinant molecules exhibiting antimicrobial properties.

## 3. Antimicrobial Potential of Endolysins Derived from Bacteriophages Infecting *Geobacillus* and *Parageobacillus*

### 3.1. Historical Perspectives

The history of phages starts in 1896 when Ernest Hankin observed the antibacterial activity of water from the Ganges against *Vibrio cholerae* [101]. Nineteen years later, Frederik Twort described the activity of phages in detail but at that time he was not sure whether he observed the activity of an “ultra-microscopic virus”, “a living protoplasm” or “an enzyme with power to growth” [102]. It was not until 1917 when Félix d’Hérelle realized that what he was observing were bacterial viruses and gave them a name—bacteriophages [102]. D’Hérelle was also the first researcher to introduce phage therapies. First, he proved phage therapy to be effective in extinguishing avian typhoid epidemics in poultry and then he shifted his focus to the application of phage therapy in humans. The first successful phage therapy in humans took place in 1919 in France, where d’Hérelle treated several children suffering from dysentery. [103]. More or less successful attempts to use phage therapies continued until after World War II, but most of the Western world abandoned research in this topic as antibiotics were discovered and seemed to be a much more promising option for antibacterial treatment [104].

Today, as we enter the so-called post-antibiotic era, with antibiotic-resistance growing faster than new antibiotics can be discovered, there is an urgent need for alternative antimicrobial treatments. Obviously, phage therapy is one such option, but as far as the phages of the *Geobacillus* or *Parageobacillus* genera are considered, they are not expected to be able to target bacteria typically responsible for infections in humans as phages tend to have a rather narrow host range. However, phages of *Geobacillus* or *Parageobacillus* genera could offer biotechnology many other opportunities to support advances in antimicrobial treatments. They could be: (1) a source of lytic enzymes—some endolysins such as SAL200 have already been tested via intravenous administration in humans [105] and some formulations like Staphefekt ™ are already products ready to be used on human skin, or (2) a platform for the presentation of antigens, i.e., vaccines—so far at least 16 bacteriophage-based vaccines have been tested on animals [106], but none of them were based on a phage of the *Geobacillus* or *Parageobacillus* genera.

### 3.2. Endolysins as Novel Antimicrobials

**Figure 3:**
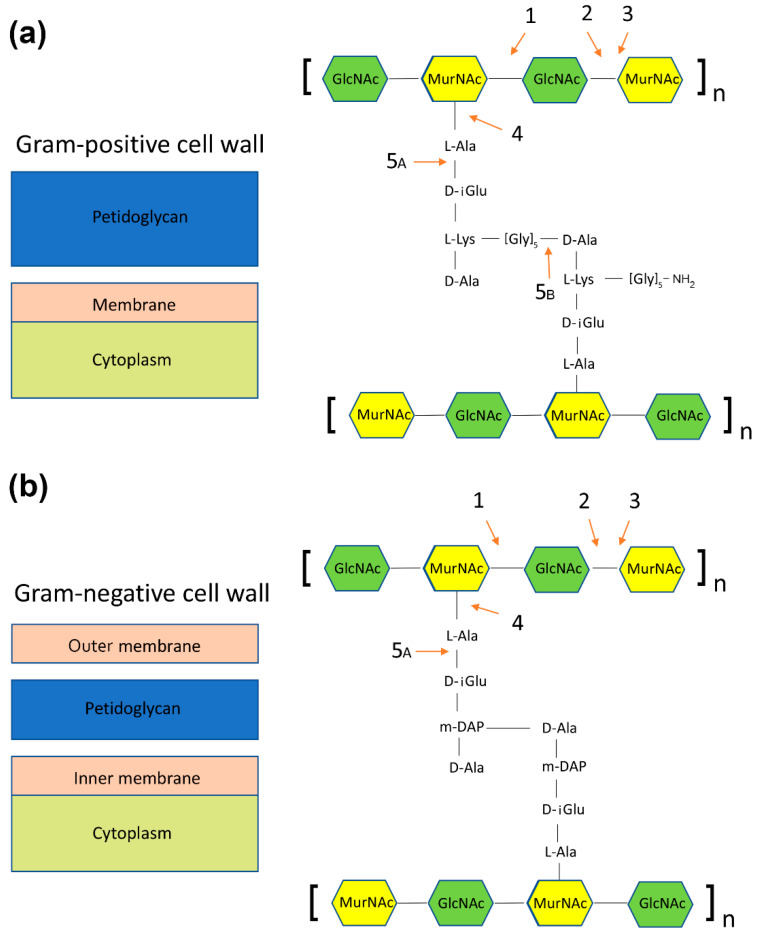
Schematic representation of the mechanism of action of endolysins on Gram-positive (panel **a**) and Gram-negative bacteria (panel **b**). (1) *N*-acetyl-beta-d-glucosaminidase; (2) *N*-acetyl-b-d-muramidase; (3) lytic transglycosylase; (4) *N*-acetylmuramoyl-L-alanine amidase, and (5) endopeptidases: 5A, L-alanoyl-d-glutamate endopeptidase; 5B, d-alanyl-glycyl endopeptidase.

Most of the bacteriophages, apart from the filamentous ones, encode proteins that allow the phages to escape the host cell via disruption of the cell wall. The proteins are supposed either to stop the synthesis of peptidoglycan or to digest it enzymatically. The latter is performed by a group of enzymes called endolysins, which can be further divided into five groups, depending on the exact type of bond being cleaved [107,108,109]. Figure 3 shows a schematic representation of the mechanisms of action of the endolysins on Gram-positive and Gram-negative bacteria:

1. *N*-acetylmuramidases (bond between *N*-acetylglucosamine and *N*-acetylmuramic acid) (Figure 3, panels a and b),
2. *N*-acetylglucosaminidases (bond between *N*-acetylmuramic acid and *N*-acetylglucosamine) (Figure 3, panels a and b),
3. Transglycosylases (bond between *N*-acetylmuramic acid and *N*-acetylglucosamine, a different mechanism than in *N*-acetylglucosaminidases) (Figure 3, panels a and b),
4. Amidases (bond between *N*-acetylomuramic acid and L-alanine) (Figure 3, panels a and b)
5. Endopeptidases:
  - Bond between L-alanine and d-glutamic acid (Figure 3, panels a and b);
  - Bond between glycine and d-alanine (Figure 3, panel a).

As endolysins digest peptidoglycan, they first of all need to access it, i.e., crossing the barrier formed by the inner membrane of a bacterium. Usually this is achieved through cooperation with another protein—a holin, which causes permeabilization of the inner membrane and allows endolysins into the periplasm. Some of the endolysins possess a signal–arrest–release sequence (SAR), which enables their anchoring to the inner membrane without the help of pinholins. However, this mode of action requires depolarization of the membrane to release and activate an endolysin. Such depolarization can be either spontaneous or caused by holins [110].

As far as the structure of endolysins is concerned, endolysins of Gram-negative specific phages are simple, globular proteins consisting of one catalytic domain. On the other hand, a modular structure is common for endolysins of Gram-positive specific phages: usually there is one binding domain responsible for the recognition of the target peptidoglycan and at least one catalytic domain. This difference in the endolysin structure (between phages specific to Gram-positive and Gram-negative bacteria) is probably caused by the lack of an outer membrane in Gram-positive bacteria and the need to prevent ill-timed digestion of other bacteria from the outside [111].

### 3.3. Thermophilic Endolysins from Bacteriophage Infected Geobacillus Species

Tail bacteriophages, with double-stranded DNA genomes, are often equipped with enzymes called endolysins, which cause lysis of the host by digestion of the peptidoglycan wall. Such bacteriophages usually dominate the environment. Endolysins lacking special signal sequences cannot cross the cytoplasmic membrane unaided. These enzymes access the peptidoglycan by large pores (called “holes”), created in the membrane by holins. Some endolysins do not require holins due to the N-terminus SAR sequence, which allows for their transport by the bacterial Sec system [107].

Endolysins have acquired high substrate specificity. They interact selectively with the potential substrate, which is the peptidoglycan layer of the cell wall of bacteria species. As previously mentioned, such a high affinity of the enzyme for the substrate is usually conditioned by the presence of the cell wall binding domain (CBD), typically located at the C-terminus of the protein. CBD recognizes and binds specific ligands (carbohydrates or teichoic acids) [112]. In addition to the CBD domain, phage endolysins also have the EAD domain, located at the N-terminus [112]. Thus far, 24 variants of the EAD domain and 13 variants of the CBD domain have been characterized [113]. The EAD and CBD domain structures relate to endolysins derived from mycobacteria and the bacteriophages infecting Gram-positive microorganisms. Gram-negative phage endolysins often lack the CBD domain [107,114].

Resistance to antibiotics of many pathogenic bacteria has become a serious clinical problem; therefore, a continuous search for new therapeutic strategies is necessary [115]. The World Health Organization (WHO) warns that, up to the year 2050, antibiotic-resistant bacteria could kill more than 10 million people. The most promising solutions are bacteriophages, phage endolysins and antimicrobial peptides.

Endolysins encoded by bacteriophages are of a great interest because of their potential as antimicrobial agents, useful for controlling bacterial infections and preventing biofilm formation. They can also be used in the case of unwanted contamination of food with opportunistic or pathogenic bacteria [115,116]. According to the literature, bacteriophages, endolysins, and antimicrobial peptides can be used in combination therapy. Such an approach negates many of the limitations of their specificity as single antimicrobial agents [116,117].

Only a few functionally known, thermostable endolysins have been isolated from *Geobacillus* spp. infecting bacteriophages so far. Here, we focus on their characteristics.

#### 3.3.1. Thermostable GVE2 Endolysin

The bacteriophage from the *Siphovirus* GVE2 family is one of a few isolated from *Geobacillus*. GVE2 is a virulent bacteriophage that infects thermophilic deep-sea *Geobacillus* sp. E263 (CGMCC1.7046), capable of growing at 45–80 °C, with an optimal temperature between 60 and 65 °C. The bacteriophage genome is 40,863 bp, 44.8% G + C, linear dsDNA with 62 ORFs. Proteomic analysis characterized six GVE2 proteins. A gene encoding endolysin was cloned, expressed in *E. coli*, purified and characterized. In addition, the holin–endolysin system was tested. Confocal microscopy data showed that GFP–endolysin aggregates in *Geobacillus* sp. E263 were infected with GVE2. The results revealed that GVE2 endolysin interacts directly with the phage holin. It was found that endolysin can interact with the host protein ABC transporter, suggesting that host proteins may be involved in the regulation of the lysis process [70,118].

*Clostridium perfringens* (*C. perfringens*) is a bacterial pathogen that causes necrotizing enteritis in poultry and livestock and is a source of food poisoning and gas gangrene in humans. With the decreasing use of antibiotics in feed, alternatives to antibiotics are needed. Bacteriophage endolysins are efficient at eliminating the pathogenic bacterial host. This type of enzyme is a potential replacement for the antibiotics that control *C. perfringens*. Animal feed is heat treated during pellet production. This treatment consists of the mixture of feed ingredients and conditioning with steam at temperatures up to 95 °C, with an exposure time from 20 s to 4 min. Thus, endolysins used in the production of animal feed pellets should be thermostable or thermotolerant. Additionally, their EAD domains can often be modified to target different bacterial species [119]. That makes the thermostable lytic enzymes highly desirable.

Swift et al. generated thermostable endolysins directed against *C. perfringens*. Thermostable, catalytic endolysin domains were fused to CBD domains derived from various *C. perfringens* prophage endolysins. Three thermostable catalytic domains were used. Two of them were prophage endolysins, identified in *Geobacillus* genomes. The third endolysin was isolated from a deep-sea thermophilic, *Geobacillus* bacteriophage E2 (GVE2). These catalytic domains carried the activities of L-alanine-amidase, glucosaminidase, and L-alanine-amidase, respectively. All were able to degrade bacterial cell wall peptidoglycan [119].

#### 3.3.2. Thermostable G05 Endolysin

*Geobacillus* bacteriophages were isolated by Micreos BV (Hague, Netherlands) and designated G01-G09. Their genomes were sequenced to determine the sequence identity among bacteriophages and to identify endolysin encoding genes. Six of the nine analyzed genomes contained easily identifiable genes coding for endolysins. Out of the six identified endolysins, three: G05, G08, G09 exhibited 99% sequence similarity. The endolysin genes were cloned into an *E. coli* expression vector and the recombinant proteins were then isolated. The lytic activity of the recombinant endolysins was determined at 50 °C, by measuring the OD at 600 nm. The recombinant enzymes showed high lytic activity against *Geobacillus* sp. in the concentration range of 2–100 µg/mL. *Geobacillus* is a bacterial organism that produces a biofilm that is strictly thermophilic and facultatively anaerobic. The authors of the patent sought a solution to remove the *Geobacillus* bacteria biofilms that interfere with the growth and processing of tobacco [120].

*Geobacillus* biofilms were found in various environments (e.g., hospitals, kitchens, bathrooms, fluid pipes, water, milk, oil, fuel, sewage, boat hulls, plants or trees, animal mouths and in paper or pulp mills). Thus, endolysins specific to the *Geobacillus* containing biofilm can be helpful in the reduction or elimination of these bacteria.

#### 3.3.3. Thermostable GVE3 Endolysin

The GVE3 bacteriophage, infecting *G. thermoglucosidasius*, is a member of the *Siphoviridae* family. Genome bioinformatics analysis revealed the presence of genes encoding lytic enzymes: endolysin and holin, but their lysing properties for host bacteria and other strains were not determined. The thermostability of both the endolysin and holin were also not investigated. However, the proteins were considered stable at high temperatures. Such endolysins could be used for example, for milk treatment, to eliminate *Geobacillus* sp. Thus, they can be of commercial value. [121].

#### 3.3.4. Thermostable TP-84_28 Endolysin

Endolysin TP-84_28 was isolated from the *G. stearothermophilus* strain 10 infected with the TP-84 bacteriophage [122]. The enzyme is thermostable. Its optimal reaction temperature is 55 °C. The protein melting point is at 77.6 °C, which was established by physicochemical analyses: differential scanning calorimetry (DSC) and circular dichroism spectroscopy (CD). Interestingly, above this temperature the enzyme still exhibits lytic activity, albeit at a lower level. Incubation of the protein at 100 °C for 30 min causes loss of activity, but not completely [Zebrowska J (unpublished)].

Although the endolysin is derived from the TP-84 bacteriophage, which infects specific *Geobacillus* strains, it also shows activity against other bacterial species. The antimicrobial effect of the endolysin was tested against two groups of bacteria: Gram-positive and Gram-negative. The enzyme was added directly to the culture of the investigated bacterial strains. Since the optimal temperature for the TP-84_28 endolysin activity is 55 °C, thermophilic bacteria were chosen for testing. The endolysin was highly active against Gram-positive bacteria such as *G. stearothermophilus*, *G. thermoleovorans*, and *Geobacillus* sp. ICI. Surprisingly, the endolysin was found to be active against mesophilic Gram-negative strains, but to a lesser extent [Zebrowska J (unpublished)].

The optimal growth temperature of mesophilic strains did not allow the testing of the endolysin activity directly in a growing microbial culture. However, simple incubation of the selected mesophilic bacteria with the endolysin at 55 °C showed its lytic activity against both Gram-positive and Gram-negative mesophilic strains, with a predominance of Gram-positive strains such as *B. cereus* and *B. subtilis* [Zebrowska J (unpublished)].

A thermostable endolysin TP-84_28 may be used for the disinfection of surfaces exposed to high temperatures or as a component of an antimicrobial wound healing preparation. Such preparations could be particularly desirable against hospital-acquired pathogenic strains.

The thermostable endolysin, isolated from the *Thermus scotoductus* MAT2119 strain infected with Ph2119 bacteriophage, is worth mentioning. The enzyme is the first type 2 *N*-acetylmuramyl-L-alanine amidase isolated from a thermophilic phage. Plotka et al. demonstrated for the first time that the thermophilic Ph2119 endolysin exhibited lytic activity against the peptidoglycan of mesophilic Gram-negative bacteria, such as: *E. coli*, *Serratia marcescens*, *Pseudomonas fluorescens* and *Salmonella enterica* serovar Panama. The Ph2119 endolysin exhibits 22% identity with the bacteriophage T7 lysozyme and 23% identity with the T3 lysozyme. The enzyme also showed similarity to the eukaryotic peptidoglycan recognition proteins involved in the innate immune defense found in both insects and mammals. This observation brings interesting conclusions leading to the possibility of using such a protein type for the recognition and digestion of peptidoglycan of eukaryotic origin [123].

Endolysins are noteworthy proteins due to their strong antimicrobial potency. A very small amount of an endolysin is able to eliminate the bacteria from a bacterial suspension. To date, there are no other biological compounds capable of killing microorganisms as quickly and effectively as endolysins. The most promising aspect of these enzymes is their ability to combat antibiotic-resistant bacteria. The efficacy of endolysins has been demonstrated against multi-drug, penicillin- and vancomycin-resistant bacterial strains. A cocktail of different endolysins, with or without antibiotics, could further enhance their antimicrobial activity. High temperatures inactivate most of the available antibiotics. Therefore, such cocktails of endolysins can be particularly useful against thermophilic bacteria [108,117].

### 3.4. Challenges in the Application of the Geobacillus Bacteriophage Derived Endolysins

Endolysins are a novel class of drugs and so their journey from the laboratory to the market poses a challenge. Thus far, only one endolysin-based product group is available on the European market: Gladskin are products for topical administration based on the active enzyme Staphefekt ™(SA.100). However, more products might be underway as research on the clinical application of various endolysins continues on endolysins such as XZ.700, Artilysin Art-175, SAL200, Exebacase, Ectolysin P128 [124].

What seems to be the biggest obstacle in the practical use of endolysins is their proteinaceous nature. Two aspects seem to be crucial: the administration route and reactions of the immune system.

Not surprisingly, the first commercially available endolysin-based products are meant for topical application—remaining on the surface of the skin, the applied protein is subject to enzymatic digestion or reaction of the immune system to a much lesser extent than when applied orally or intravenously. As far as the oral delivery route is considered, it could most probably only be used for targets within the digestive tract, because proteins are believed not to be absorbable in relevant amounts by enterocytes if not digested to peptides [125]. If an endolysin was expected to act within the digestive tract, highly variable conditions inside it have to be born in mind. The pH varies from highly acidic in the stomach to 7, 4 in the terminal ileum [126] and an inappropriate pH could readily deactivate an enzyme. Even if the influence of gastric acid is omitted via encapsulation in gastro-resistant capsules, an endolysin could be digested by enzymes such as trypsin, chymotrypsin or carboxypeptidase within the intestine. Additionally, interaction with products of the gut microbiota metabolism or ingested food could affect the activity of such enzymatic drugs as endolysins. Another possible mode of delivery of a proteinaceous drug is intravenous administration. It offers a much better bioavailability than oral intake; however, it severely limits accessibility of such a treatment to patients. Inserting a peripheral venous catheter is a simple medical procedure but requires a patient to see medical professionals for each dose of a drug to be administered. Proposed delivery routes such as nasal route and inhalation route have their own limitations, too: probably a high proteolytic activity of the enzymes of macrophage origin in the lungs and size limit absorption of a protein via mucosa in the nose [124].

As all proteins, endolysins could evoke the reaction of the human immune system. Three theoretically expected challenging phenomena could be: allergic reactions, neutralization of drug particles by raised antibodies, and the formation of auto-antibodies. Until now, there are little data on clinical trials in humans, but one worth mentioning is the human safety evaluation of endolysin SAL200 by Jun et al. [127]. Although there were no serious adverse effects including severe allergic reactions in this study, the emergence of antibodies against SAL200 was confirmed in 37% of the study group. The level of serum antibodies seemed to be proportional to the dose. An interesting observation has been made that the blood antibacterial activity was much lower than expected based on the concentration of the drug in blood and antibacterial activity of the blood samples varied between samples with similar serum concentrations of the drug. The authors indicate a possible reason for this phenomenon related to technical issues, but the presence of antibodies should also be taken into consideration as a factor for lowering the enzyme activity. Thinking about immune reactions, one should also bear in mind the possibility of the emergence of auto-antibodies and triggering of auto-immune diseases if an administered endolysin is similar to a protein present in humans. The tendency to evoke antibodies formation will vary among endolysins greatly, depending on their structure; therefore, the abovementioned issue will have to be considered for each drug separately.

Challenges that might arise due to the use of endolysins derived specifically from *Geobacillus* phages include their temperature optimum and substrate specificity. As *Geobacilli* are thermophilic, endolysins derived from phages of these bacteria can be expected to have their temperature optimum above the temperature of a human body. Though unfavorable for use in humans, higher temperature optima do not exclude industrial applications. Another factor limiting the use of endolysins derived from *Geobacillus* phages is their affinity for bonds found in the peptidoglycan of these specific bacteria. If similar structures of peptidoglycan are present in another bacterial strain or genus, the activity of an endolysin will be present. Therefore, the experimental testing of endolysins against various bacterial strains might be required to confirm their activity.

## 4. Outlook for Using Antimicrobials Derived from *Geobacillus*, *Parageobacillus*, and Their Bacteriophages

The age of antibiotics in the fight against pathogenic microorganisms is almost over, mainly due to the increasing antibiotic resistance of contemporary pathogens. This situation causes a rapidly growing interest in antimicrobial compounds that could be alternatives to antibiotics. The *Geobacillus* and *Parageobacillus* genera, as well as their bacteriophages, could offer many antagonistic compounds (such as: bacteriocins, probiotics, postbiotics, or endolysins) with a wide range of biological functions. Currently, the bacteriocins seem to be the best-studied group of the antimicrobial substances. However, their routine use requires further research, especially to improve their stability, bioavailability, solubility and performance in vivo. The *Geobacillus* and *Parageobacillus* derived bacteriocins may help to solve several problematic issues. Such thermostable antimicrobial compounds could be particularly useful in the food industry, in veterinary treatment, or to disinfect surfaces exposed to high temperatures. Additionally, screening for novel, beneficial environmental strains with probiotic qualities within the *Geobacillus* and *Parageobacillus* genera could be a promising future trend in new probiotic preparations. The industrial and environmental interest in these genera is worth greater investigation, especially given their bio-safety, resistance properties, and their wide range of action against mesophilic pathogenic bacteria. Both genera are largely unexplored and could reveal new functionalities and products of medicinal or industrial value. However, it should be noted that in some cases there is still a lack of specific and concrete data on *Geobacillus*/*Parageobacillus* derived antimicrobial compounds, making it difficult to assess the true antimicrobial potential of these bacteria.

## Figures and Tables

**Table 1:** Characterized and semi-characterized bacteriocins from the *Geobacillus* and *Parageobacillus* species.

Bacteriocin	Parent Strain	Determined Antimicrobial Bioactivity against Mesophilic Strains	Properties	Reference
geobacillin I	*G. thermodenitrificans* NG-80-2 *G. thermodenitrificans* DSM465 *G. thermodenitrificans* OHT-1 *Geobacillus* sp. M10EXG *G. thermodenitrificans* OH2-1 *G. thermodenitrificans* OH5-2	*Streptococcus dysgalactiae* Vancomycin-resistant *Enterococcus faecium* Methicillin-resistant *S. aureus* *B. anthracis* Sterne 7702	nisin analog; the N-terminal structure resembles nisin rings; the mode of action includes the formation of the pores in the cell membrane; comparable bioactivity range to nisin; higher pH and temperature stability than nisin;	[42]
geobacillin II	*G. thermodenitrificans* NG-80-2 *Geobacillus* sp. G11MC16	*B. cereus* Z4222 *B. subtilis*	nisin analog; the N-terminal structure resembles nisin rings; antimicrobial activity towards *Bacillus* spp. only;	[42]
toebicin 218	*P. toebii* strain HBB-218	*B. coagulans* DSM 1	ND	[44]
thermoleovorin-S2	*G. thermoleovorans* S-II	*Streptococcus faecalis (S. faecalis)* *S. typhimurium* *Branhamella catarrhalis*	high molecular weight bacteriocin: 42 kDa; stability over a wide pH (pH 3–10); activity at 60 °C and at 70 °C; partial activity at 80 °C; antimicrobial activity towards *G. thermoleovorans* (except the host strain)	[45]
thermoleovorin-N9	*G. thermoleovorans* NR-9	*S. faecalis* *S. typhimurium* *Branhamella catarrhalis*	high molecular weight bacteriocin: 36 kDa; stability over a wide pH range (pH 3–10); activity at 60 °C; antimicrobial activity towards *G. thermoleovorans* (except the host strain);	[45]
geobacillin 26	*G. stearothermophilus* 15	ND	stable at 50 °C only for 2 h; probable mesophilic origin or HGT; no effect on the tested *Candida* and *Staphylococcus* strains; may belong to non-lytic bacteriocins and its mode of action may require the binding to external cell receptors and dissipation of the membrane receptors; antimicrobial activity towards *Geobacillus* and *Parageobacillus* strains only;	[46]
thermocin 32A	*G. stearothermophilus* 32A	*B. cereus DSM 12001* *S. haemolyticus P903*	low molecular weight bacteriocins: 5.6–7.2 kDa; activity towards a closely related strain of *Geobacillus*; extremely high thermostability (no activity loss observed after incubation at 100 °C); activity at the pH range 4–10;	[17]
thermocin 17	*G. stearothermophilus* 17	*B. cereus DSM 12001*		
thermocin 30	*G. stearothermophilus* 30	*S. haemolyticus* P903 *P. aeruginosa* ATCC 27853		
thermocin 93	*G. stearothermophilus* RS93	*B. subtilis ATCC 10783*	molecular weight: 13.5 kDa; antimicrobial activity towards ten *Geobacillus* strains; optimal activity at pH 7; no activity loss observed after incubation at 70 °C; the mode of action could not be elucidated due to variable results;	[47]
thermocin 10	*G. stearothermophilus* NU-10	*B. circulans* 4516	low molecular weight: 20 kDa; remains active in the pH range 2–12 at 70 °C; the mode of action probably linked with RNA inactivation or cell membrane structure impairing;	[48,49]
unnamed bacteriocines ^1^	101 strains of *Geobacillus* spp. from the culture collection of the Department of Microbiology and Biotechnology of Vilnius University	*Streptococcus pyogenes ATCC 19615* *Streptococcus pneumoniae ATCC 6305* *E. faecalis ATCC 2912* *Enterococcus faecium 402-3/03* *Haemophilus influenzae ATCC 10211* *S. aureus ATCC 25923* *S. haemolyticus P903* *S. epidermidis ATCC 12228* *P. aeruginosa ATCC 27853* *E. coli ATCC 25922* *Klebsiella pneumoniae DSM 30104* *Yersinia enterocolitica ATCC 9610* *S. typhimurium ATCC 14028* *S. enteritidis ATCC 13076* *Listeria monocytogenes ATCC 19117* *Listeria innocua ATCC 33090* *B. cereus DSM 12001* *B. subtilis ATCC 6633* *Clostridium perfringens ATCC 13124*	ND	[50]

^1^ All of the tested strains were able to inhibit at least one of the listed, mesophilic strains; ND—not determined; HGT – horizontal gene transfer.
